# Supplementary material for: Genomic diversity of SARS-CoV-2 can be accelerated by mutations in the nsp14 gene
Source: iScience. 2023 Feb 16;26(3):106210. doi: 10.1016/j.isci.2023.106210 (PMC9933857; doi:10.1016/j.isci.2023.106210)
Supplement: Document S1. Figures S1–S7 [file mmc1.pdf]

iScience, Volume *26*

Supplemental information

Genomic diversity of SARS-CoV-2 can be accelerated by mutations in the nsp14 gene

Kosuke Takada, Mahoko Takahashi Ueda, Shintaro Shichinohe, Yurie Kida, Chikako Ono, Yoshiharu Matsuura, Tokiko Watanabe, and So Nakagawa

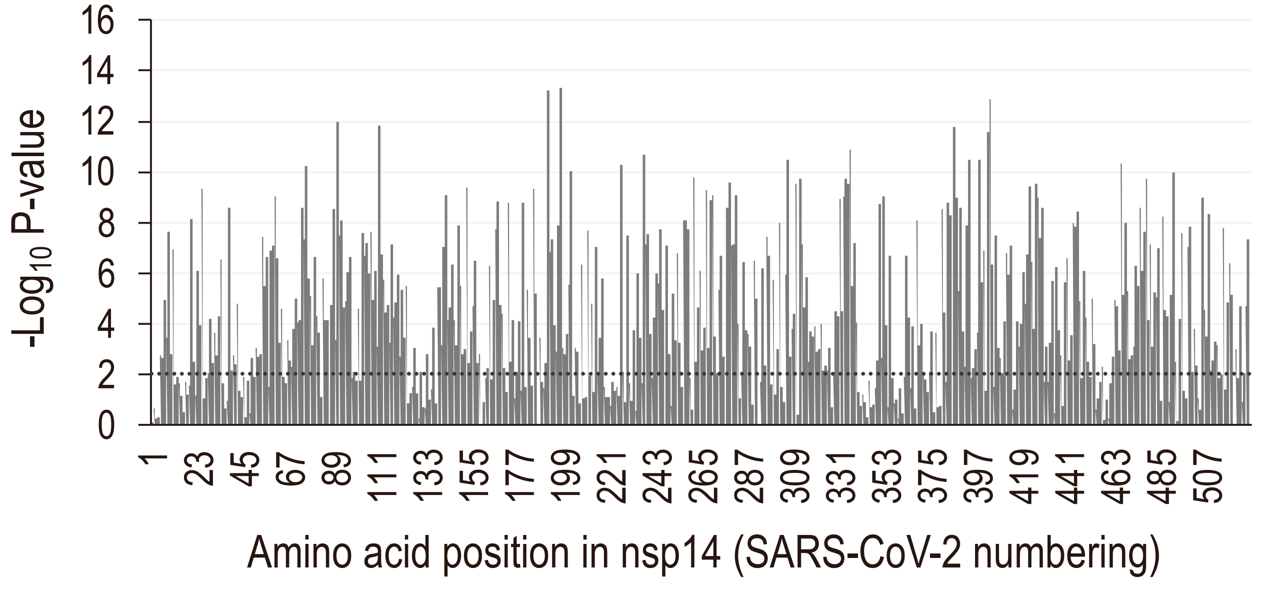

Supplementary Figure 1. Negative selection profiles of nsp14 encoded by the 62 representative coronavirus genomes, Related to Figure 1

The abscissa indicates the codon positions, with the scale bar at the top. The ordinate indicates the ($-\log_{10}$ P) value for each position when dN/dS < 1. Each position of nsp14 is shown according to the numbering of SARS-CoV-2. The codon position 504 of nsp14 was calculated by using 61 CoVs excluding the Chinese waterside skink CoV. Dotted line indicates $P = 0.01$.

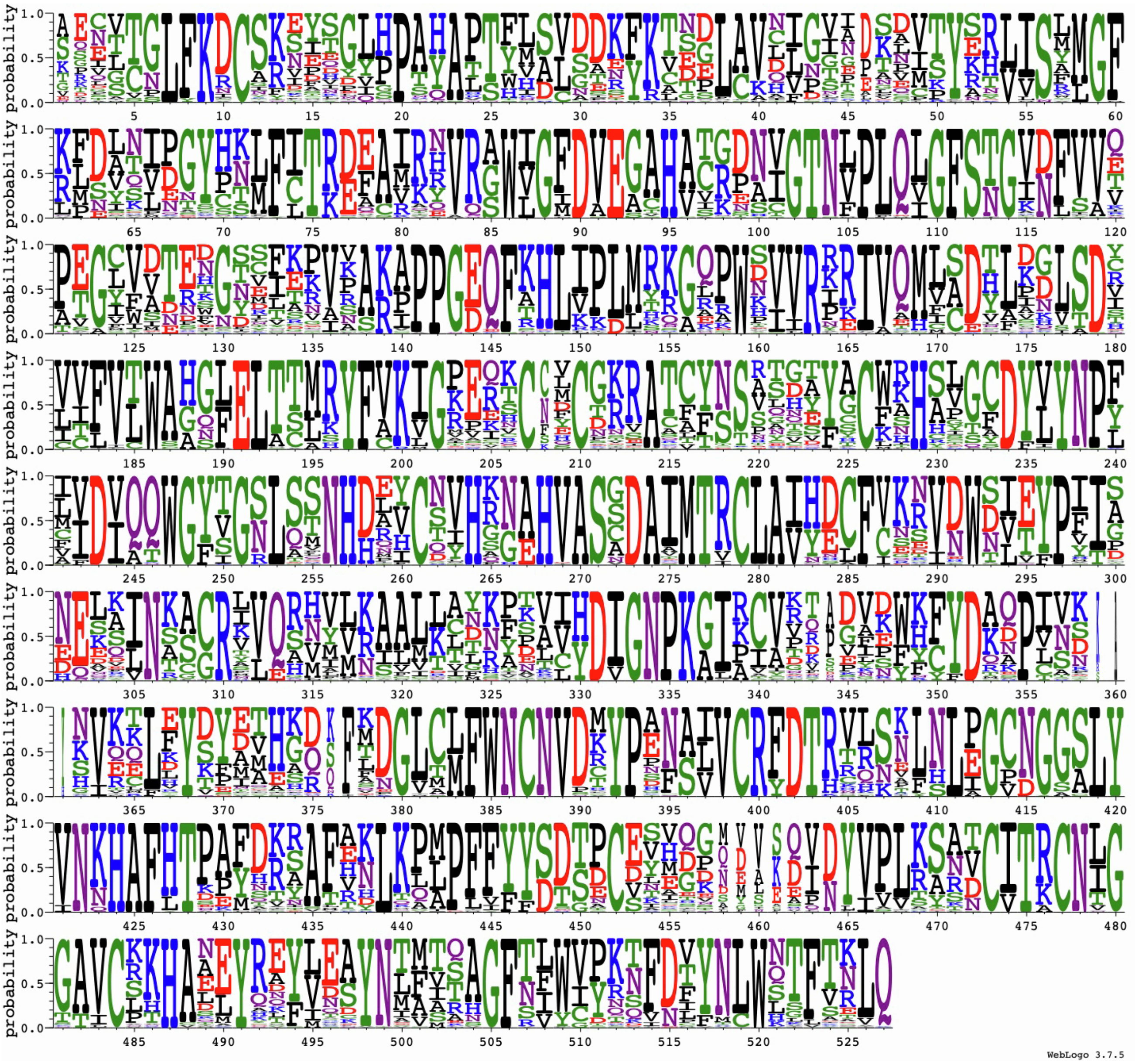

Supplementary Figure 2. WebLogo highlighting conserved amino acids in the nsp14 of 62 representative coronaviruses, Related to Figure 1C

From the sequence alignment of the nsp14 of the 62 representative coronaviruses, the amino acids aligned at the amino acid positions of SARS-CoV-2 are shown.

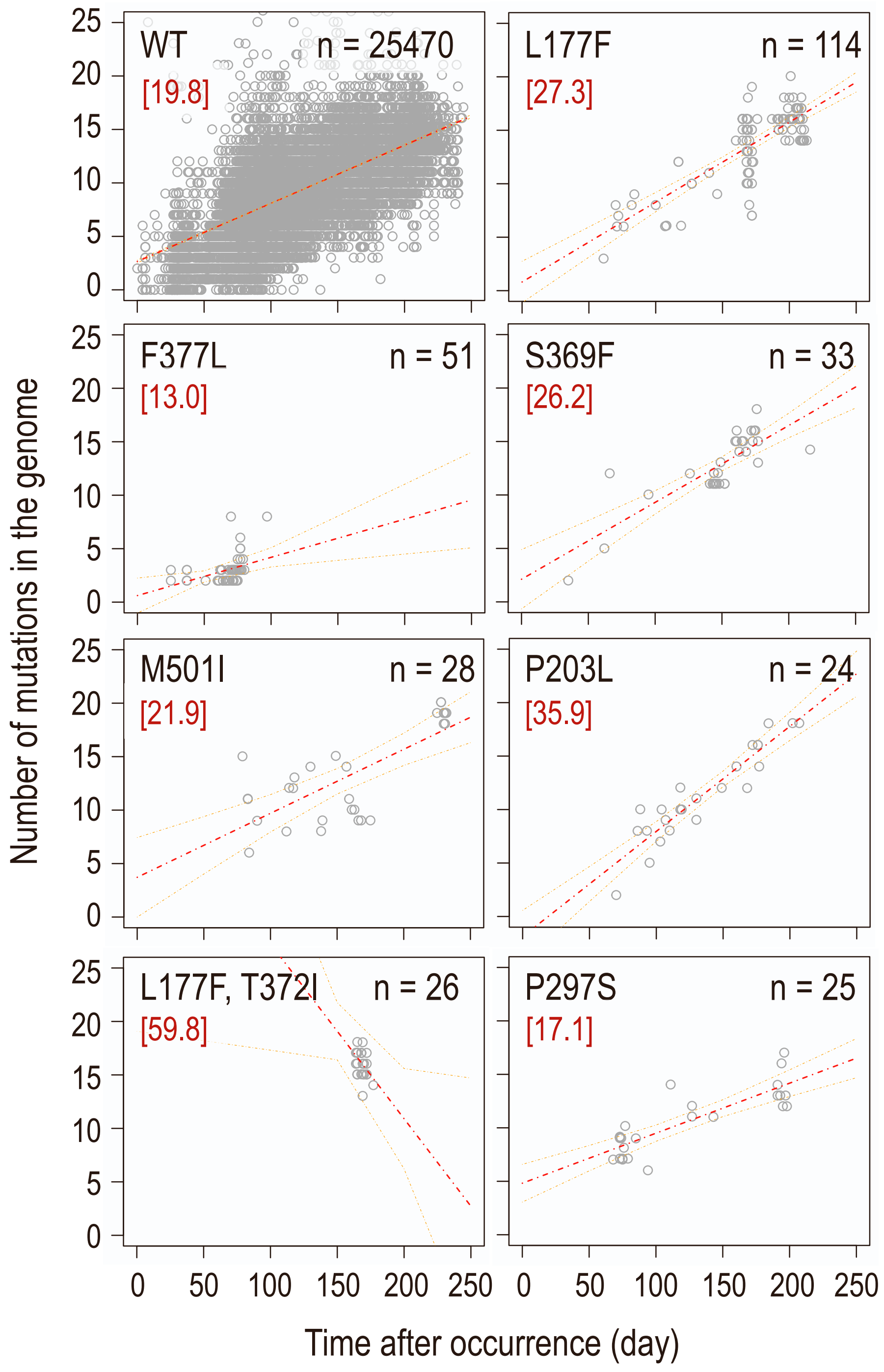

Supplementary Figure 3. Scatter plot of nucleotide mutations in SARS-CoV-2 genomes, Related to Table 2

Genome diversity of SARS-CoV-2 containing wild-type (WT) nsp14 and 7 nsp14 mutants is shown. The X-axis indicates the sampling date and the Y-axis indicates the number of nucleotide mutations in the genomes. Mutation rates per year in the genome are shown in red letters. The red and orange dotted lines correspond to the regression line and 95 % confidence intervals, respectively.

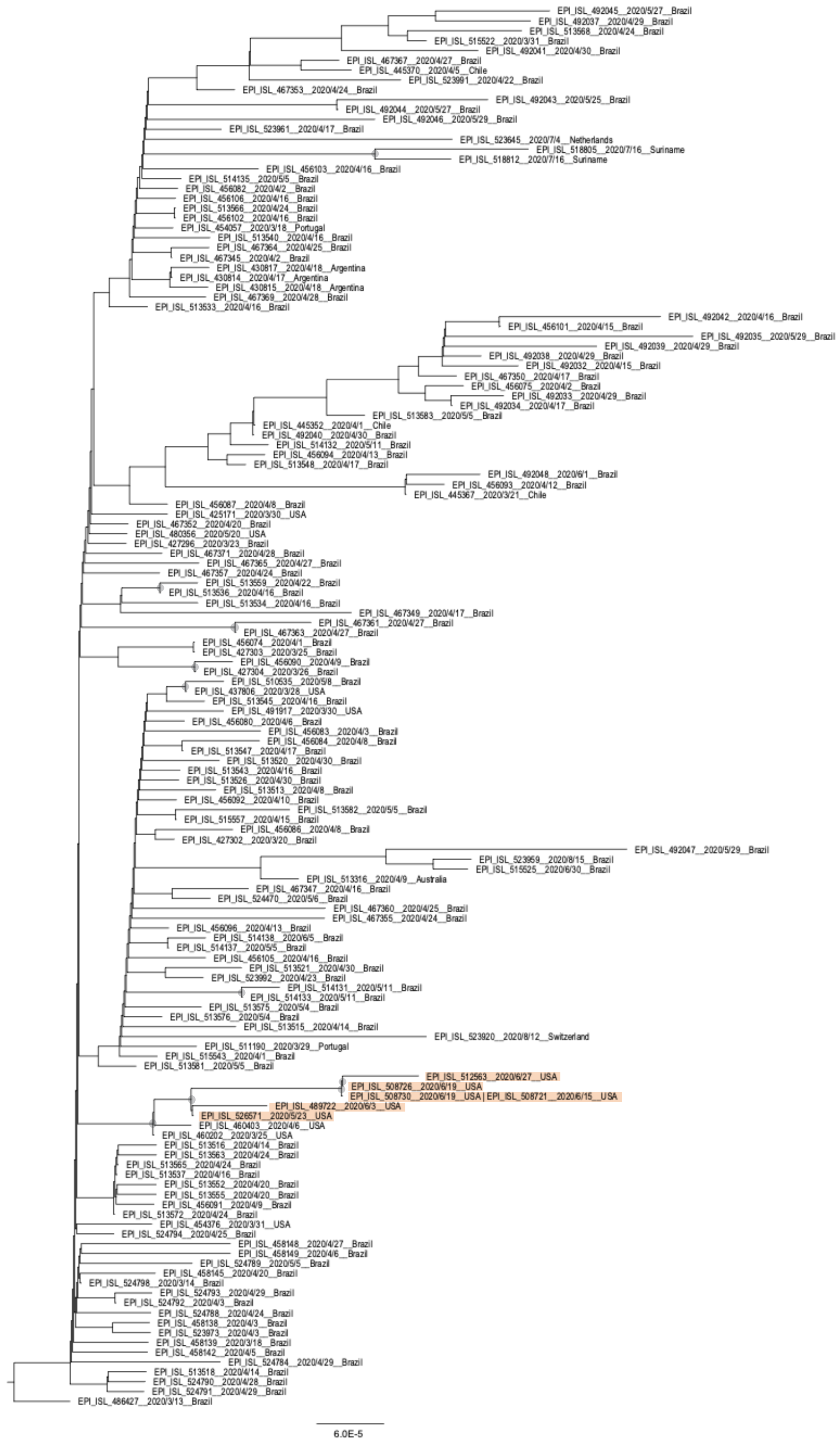

Supplementary Figure 4. Phylogeny of nsp14-P203L variants in the PANGO lineage B.1.1.33, Related to Figure 2A

ML tree of SARS-CoV-2 genomes in the B.1.1.33-lineage. The nsp14-P203L variant is highlighted in light red. A gray circle corresponds to bootstrap values ≥ 70%.

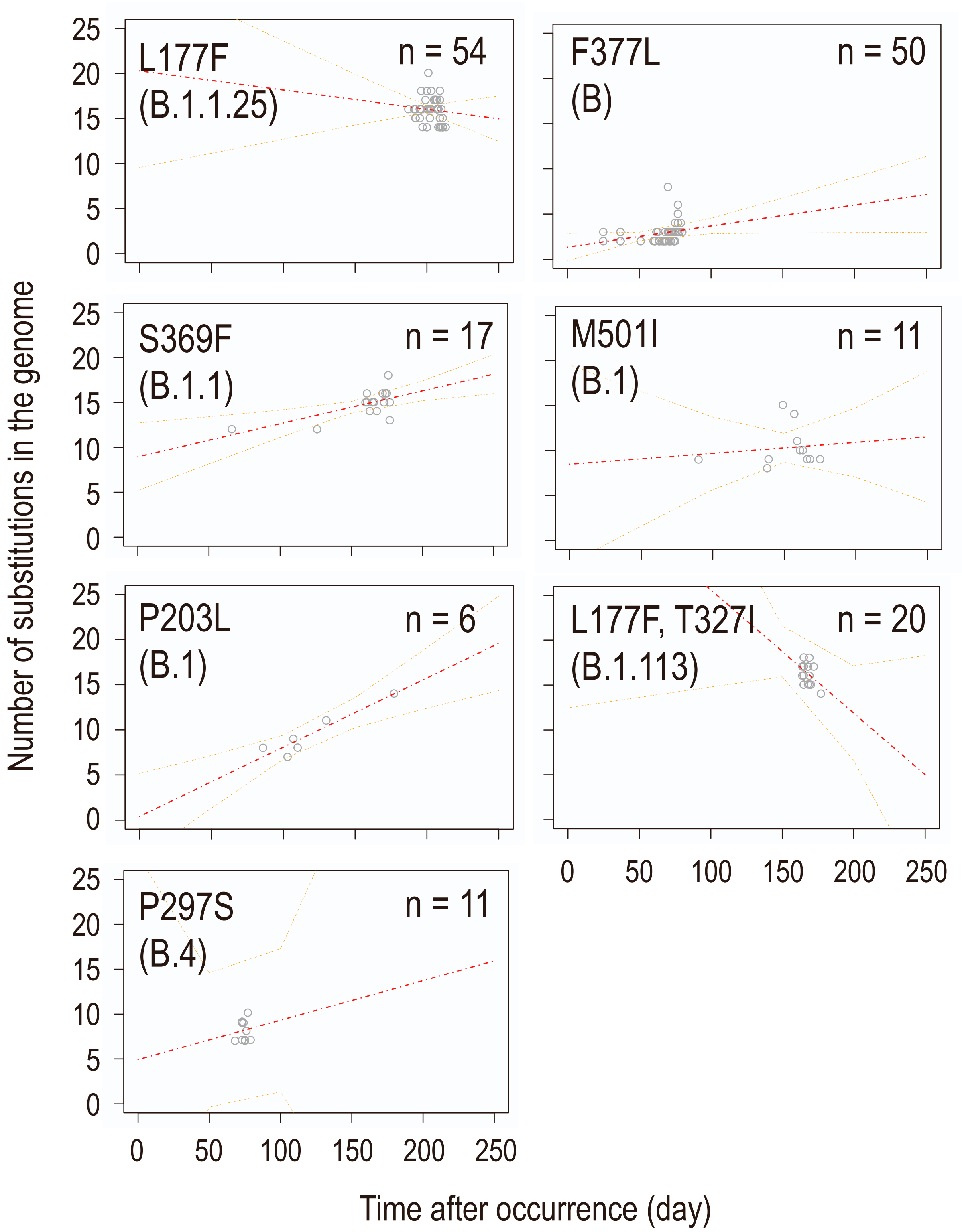

Supplementary Figure 5. Scatter plot of the 7 nsp14 variants representing each cluster where the maximum number was observed, Related to Table 3

Nucleotide mutation rates of 7 nsp14 variants for each cluster. The X-axis indicates the sampling date and the Y-axis indicates the number of nucleotide mutations in the genomes. The red and orange dotted lines correspond to the regression line and 95 % confidence intervals, respectively.

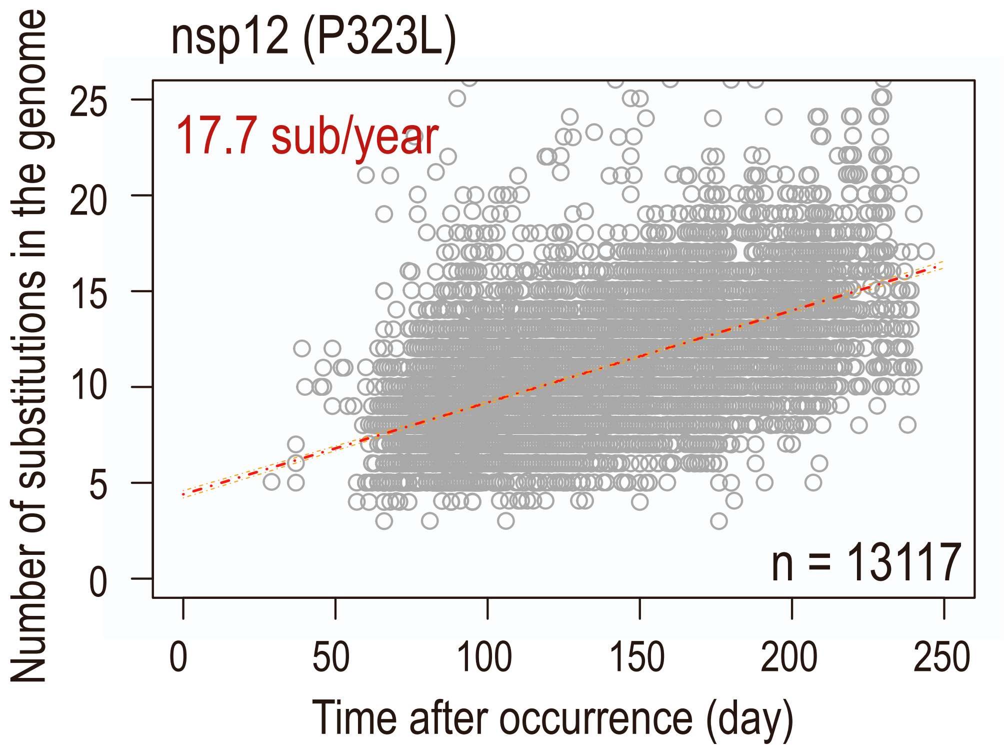

Supplementary Figure 6. Scatter plot of nucleotide mutations in nsp12-P323L variants, Related to Figure 2B

Genome diversity of nsp12-P233L variants is shown. Duplicate sequences were removed, leaving only the sequences with the oldest sampling date. The X-axis indicates the sampling date and the Y-axis indicates the number of nucleotide mutations in the genomes. Mutation rates per year in the genome are shown in red letters. The red and orange dotted lines correspond to the regression line and 95 % confidence intervals, respectively.

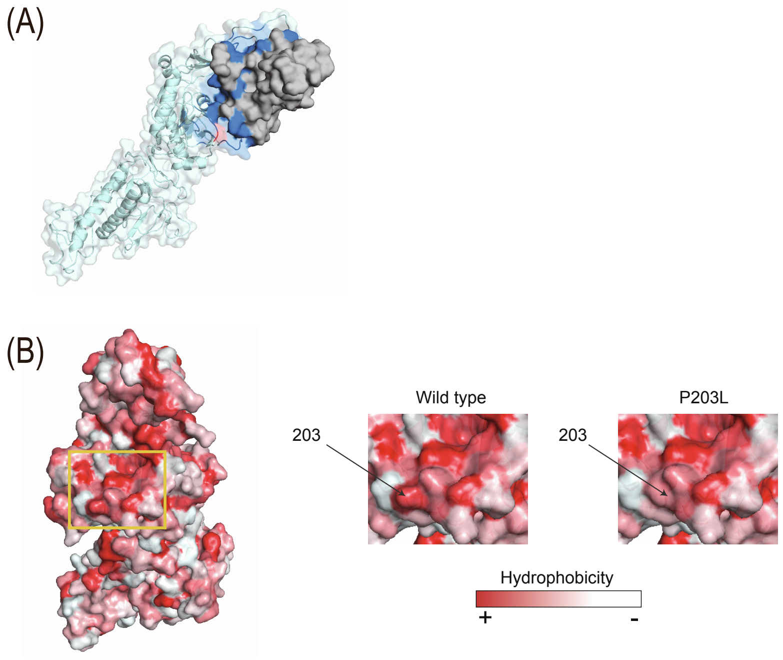

Supplementary Figure 7. Three-dimensional position of the P203L mutation in nps14 and the interaction site with nsp10, Related to Figure 3

(A) Amino acid position on the three-dimensional structure of nsp14. The nsp14 and nsp10 is shown in light blue and gray, respectively. The site of interaction with nsp10 is shown in dark blue. Amino acid position 203 in nsp14 is shown in red. (B) Hydrophobic (red) and hydrophilic regions (white) at the surface of the nsp14-203P (wild type) or nsp14-203L protein.
